# Impact of laterality and mucinous histology on relapse-free and overall survival in a registry-based colon cancer series

**DOI:** 10.1038/s41598-019-40096-6

**Published:** 2019-03-06

**Authors:** Francesca Negri, Annamaria De Giorgi, Annalisa Gilli, Cinzia Azzoni, Lorena Bottarelli, Letizia Gnetti, Matteo Goldoni, Laura Manotti, Paolo Sgargi, Maria Michiara, Francesco Leonardi, Guido Rindi, Stefano Cascinu, Enrico Maria Silini

**Affiliations:** 1 Medical Oncology Unit, University Hospital of Parma, Parma, 43126 Italy; 2 Department of Medicine and Surgery, Unit of Pathological Anatomy, University Hospital of Parma, Parma, 43126 Italy; 3 Medical Statistics, Department of Medicine and Surgery, University of Parma, Parma, 43126 Italy; 4 Pathology Unit, Istituti Ospitalieri di Cremona, Cremona, 26100 Italy; 5 Institute of Anatomic Pathology, Catholic University, Rome, 00168 Italy; 6 Department of Medical and Surgical Sciences for Children and Adults, Division of Medical Oncology, Policlinico di Modena Azienda Ospedaliero-Universitaria di Modena, Modena, 41124 Italy

## Abstract

Recent data suggest that tumor laterality and mucinous histology may be clinically relevant. We investigated how both variables impact on the prognosis and the response to therapies in a large population-based cohort of cancer patients. Incidence data, clinical and pathological features, and outcome were systematically collected from the Tumor Registry of Parma over the years 2004–2009. Survival data were modeled by multivariable analysis. 1358 patients affected by stage I–IV colon cancer were considered; 661 (49%) had right-sided and 697 (51%) left-sided tumors. 144 (11%) had mucinous (MAC) and 1214 (89%) non-mucinous (NMAC) histology. MACs and NMACs of the right colon showed no difference in stage distribution, whereas left colon MACs were more frequently in an advanced stage (stage IV) (p = 0.008). Stage IV right colon tumors had a poorer overall survival than stage IV left-sided colon cancers (75^th^ percentile 20 vs 34 months, p < 0.001). At relapse, MACs were less responsive to systemic therapy and had worse survival compared with NMACs regardless of tumor side (7.1 vs 13.1 months, p = 0.018). Right-sided colon cancers had poorer survival compared to left-sided tumors; the effect was mainly attributable to NMACs. At relapse, MACs had unfavorable prognosis regardless of the primary tumor-side.

## Introduction

Colorectal cancer (CRC) is the third most common tumor and the fourth leading cause of cancer-related deaths worldwide^1^. Clinical behavior and prognosis of CRC depend mainly on tumor stage, grade, age, gender, molecular and pathological features^2^.

Two controversial issues recur in CRC literature, the effect of laterality (proximal vs distal CRC)^3–6^ and the role of mucinous histology^7,8^. The two issues partly overlap as mucinous CRC tend to occur more frequently in the proximal colon^7,8^. A dichotomy between right-sided (proximal to the splenic flexure) and left-sided CRC (distal to the splenic flexure) is supported by embryological, epidemiological, clinical, pathological and molecular data^5^. Laterality has been reported as an independent prognostic and predictive factor in many studies^3–6,9–12^, although few of them have relied on population-based data^4^. A different distribution in molecular subtypes likely underlies the variable behavior of proximal and distal CRC^13,14^, nonetheless laterality is an effective clinical proxy.

Mucinous adenocarcinomas (MACs) are tumors with >50% of extracellular mucin^15^ and account for 4–15% of all CRCs with variable rates among geographical areas, higher in Western than in Asian countries^16^. MAC is a predictor of poor outcome^16–18^, but it is contentious whether histology exerts an independent effect or it is dependent on site, stage or genetic features such as MSI (microsatellity instability)^19,20^. MSI-high MACs are usually low-stage tumors with good prognosis, but metastatic and recurrent MACs behave aggressively and are unresponsive to therapy^21^.

In the present study, we addressed these issues on a population-based series of 1358 CRC cases derived from the Tumor Registry of the Parma Province over the period 2004–2009. The study included pathological revision of all cases with an original diagnosis of MAC and the analysis of the effect of therapy on both primary and recurrent tumors.

## Materials and Methods

The study base were all consecutive patients with a diagnosis of CRC recorded in the Parma Province Tumor Registry between 1^st^ January 2004 and 31^th^ December 2009. The Tumor Registry in Parma is accredited by IARC (International Agency for Research on Cancer) and AIRTUM (Associazione Italiana Registro Tumori); it covers a population of 423295 inhabitants and is active since 1978. Most residents (95%) are diagnosed and treated locally and refer to a single Oncology and Pathology Service.

The choice of the study period accounts for differences in treatment due to the introduction in 2004 of oxaliplatin and irinotecan in addition to fluorouracil both in the adjuvant and metastatic setting^22–24^. Moreover, since that date, antiangiogenetic or anti-Epidermal Growth Factor Receptor (EGFR) agents have entered the clinical practice often in combination with chemotherapy^25^. The accrual was stopped in 2009 to guarantee a minimum follow-up time of 5 years (median, 66 months, IQ range, 25–79 months).

Right-sided (proximal) CRCs were tumors located in the cecum, ascending, hepatic flexure and transverse colon; left-sided (distal) CRCs were distal to the splenic flexure. All histological slides of tumors with an original diagnosis of mucinous adenocarcinomas or of adenocarcinomas with a mucinous component were reviewed by an experienced pathologist to avoid misclassification based on the WHO 2010 criteria^15^. Signet-ring cell adenocarcinomas, neuroendocrine tumors, medullary and undifferentiated histotypes were excluded as well as tumors originating from the appendix and the rectum. Patients with second tumor histories were also excluded.

Clinical information was gathered from radiological exams, folders, hospital discharge forms, and general practitioner contacts. Collected data included sex, age of diagnosis, tumor side, post-diagnosis progression, metastasis sites, number of metastasis, adjuvant and/or metastatic treatments, and local therapies (surgery and/or radiofrequency). Data on chemotherapy schedules were also obtained. The schedules of chemotherapy were composed by a combination of drugs including fluorouracil/capecitabine, oxaliplain, irinotecan, mitomycin, bevacizumab, cetuximab and panitumumab according to the setting of disease and enrollment in a clinical trial.

No evidence of disease was defined as absence of metastatic or persistent tumor. Multiple metastatic deposits in a single extra-colic site were considered a single metastasic site. The outcome of first-line chemotherapy was assessed by radiology according to RECIST 1.1 criteria (Response Evaluation Criteria in Solid Tumors)^26^. If relapse occurred within the first 4 months after diagnosis, the disease was included in *de novo* metastatic group. For statistical purposes, cases with a 5-year post-diagnosis relapse time were not considered in an advanced stage.

This is a retrospective observational study based on cases collected routinely by the Parma Province Tumor Registry. The registry is accredited by IARC (International Agency for Research on Cancer) and AIRTUM (Associazione Italiana Registro Tumori) and operates in accordance with the recommendations of the Italian Data Protection Authority (Garante per la protezione dei dati personali) (Italian Garante della Privacy D.D.L. A.S. 2935). For this type of study formal consent is not required according to the Italian Data Protection Authority (Garante per la protezione dei dati personali) (Italian Garante della Privacy D.D.L. A.S. 2935). The conceptualization and design of the research was settled and agreed with the administrator responsible for Tumor Registry and approved by the Institutional Review Board (IRB), Parma Province Tumor Registry.

### Statistical analysis

Age was reported as median (interquartile range) due to non-normality and differences between two groups were tested by means of Mann-Whitney test. All other categorical variables were reported as absolute and relative frequency. Differences between categorical variables were assessed by chi-square or Fisher’s exact test as appropriate. Survival analysis was calculated by Kaplan-Meier curves and the Log-rank test. Multivariate models of Cox’s regression were used to test the overall significance of the prognostic factors, by using the variables and their product with log (time) one by one or all together to test the proportional hazard assumption. SAS 9.4 (SAS institute, Cary, NC, USA) and SPSS 23 (IBM, Amork, NY, USA) statistical softwares were used for the analysis. A p value = 0.05 was considered significant.

Propensity score matching was performed by using the FUZZY Python-based extension for SPSS (logistic regression model). The match tolerance was set at 0.02, which permitted a high number of valid cases (n = 1130), but eliminating the statistical differences due to the predictors (gender, age at diagnosis, mucinous histology and grade) between right- and left-sided colon cancer (selection variable/outcome).

## Results

The original database included 1619 patients with a diagnosis of stage I to IV CRCs. After exclusion of patients lacking follow-up data (n = 83), stage (n = 70), grade (n = 99), and unknown site (n = 9), the final number of cases was 1358. Their main characteristics are summarized in Table 1. Overall, 661 (49%) patients had right-sided and 697 (51%) had left-sided tumors. Non-mucinous histology (NMAC) was found in 1214 (89%) patients whereas 144 (11%) had a diagnosis of MAC after review. 30% of colon cancers originally recorded as mucinous did not meet the 2010 WHO criteria and were reclassified.

**Table 1:** Clinical and pathological features.

Variable	
Age at diagnosis (years)	72.5 (63.0–79.0)
Gender	Male: 748 (55%)
	Female: 610 (45%)
Side	Right: 661 (49%)
	Left: 697 (51%)
Histology	MAC: 144 (11%)
	NMAC: 1214 (89%)
T	1: 126 (9%)
	2: 210 (16%)
	3: 807 (59%)
	4: 201 (15%)
	NA: 14 (1%)
N	0: 782 (58%)
	1: 303 (22%)
	2: 227 (17%)
	NA: 46 (3%)
M	0: 1161 (85%)
	1: 174 (13%)
	NA: 23 (2%)
Stage	I: 286 (21%)
	II: 469 (34%)
	III: 376 (28%)
	IV: 227 (17%)
Grade	1: 180 (13%)
	2: 709 (52%)
	3: 469 (35%)
Excised Lymph nodes	18 (13–24)
Positive Lymph nodes	0: 769 (57%)
	1: 147 (11%)
	>1: 382 (28%)
	Not Available: 60 (4%)
Metastasis site at the diagnosis (total)	Liver: 165 (12%)
	Lung: 29 (2%)
	Lymph nodes: 21 (2%)
	Peritoneum: 63 (5%)
	Other: 44 (3%)
Number of metastatic sites at the diagnosis	1: 163 (12%)
	>1: 69 (5%)
Metastasic site at relapse (187 patients)	Liver: 107 (57%)
	Lung: 42 (22%)
	Lymph nodes: 30 (16%)
	Peritoneum: 42 (22%)
	Other: 65 (35%)
Number of metastatic sites at relapse	1: 105 (56%)
	>1: 82 (44%)
Administration of first line chemotherapy	203 (15%)
Stage distribution in chemotherapy-treated patients	I: 7 out of 279 (2%)
	II: 27 out of 442 (6%)
	III: 51 out of 325 (14%)
	IV: 118 out of 227 (52%)

MAC, Mucinous adenocarcinomas; NMAC, Non-mucinous adenocarcinomas.

### Univariate analysis and interactions between predictors

**Figure 1:**
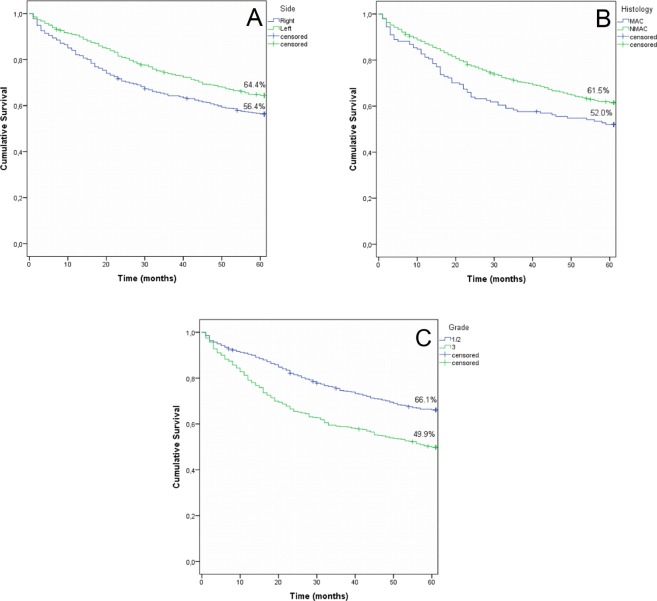
(**A**) Overall survival by tumor side. Blue = right colon, green = left colon (p < 0.001). (**B**) Overall survival by histology. Blue = mucinous adenocarcinoma, green = non-mucinous adenocarcinoma (p = 0.009). (**C**) Overall survival by differentiation grade. Blue = grade I and II, green = grade 3 tumor (p < 0.001).

Right-sided CRCs had a worse OS compared with left-sided tumors (75^th^ percentile 20 vs 34 months, p < 0.001; Fig. 1A). Mucinous histology (75^th^ percentile 16 vs 29 months; p = 0.009, Fig. 1B) and grade 3 (75^th^ percentile 16 vs 36 months, p < 0.001, Fig. 1C) were also associated with a worse prognosis. MACs did not show any difference in outcome according to side (data not shown).

Laterality was significantly related to stage, grade and histology at diagnosis (Table 2). Compared with left-sided CRCs, patients with right-sided tumors had a lower prevalence of stage I (16% vs 26%, p < 0.001) and a higher prevalence of grade 3 (42% vs 28%, p < 0.001). Seventeen percent of the patients in both right-sided and left-sided groups had stage IV disease. Histology was related to tumor stage, grade and site. In fact, MACs were more frequent in the right than in the left colon (64% vs 36%; p < 0.001) and were diagnosed at a more advanced stage compared with NMACs (stage IV 23% vs 16%, p = 0.003). Especially in the left colon MACs were diagnosed at higher stages (stage I MAC 10% vs NMAC 27%; stage IV MAC 27% vs NMAC 16% p = 0.008, whereas NMACs were more advanced in the right than left colon (stage I 17% vs 27%; p < 0.001). Laterality was also related to the age at diagnosis with similar trends in MAC and NMAC [median age: 76 (right) vs 71 (left) years, p = 0.045 in MAC; 74 (right) vs 70 (left) years, p < 0.001 in NMAC].

**Table 2:** Relationship between predictive factors at diagnosis.

	Side: Right	Side: Left	Significance
Stage	I: 106 (16%)	I: 180 (26%)	p < 0.001
	II: 252 (38%)	II: 217 (31%)	
	III: 193 (29%)	III: 183 (26%)	
	IV: 110 (17%)	IV: 117 (17%)	
Grade	1 or 2: 386 (58%)	1 or 2: 503 (72%)	p < 0.001
	3: 275 (42%)	3: 194 (28%)	
	**MAC**	**NMAC**	
Side	Right: 92 (64%)	Right: 569 (47%)	p < 0.001
	Left: 52 (36%)	Left: 645 (53%)	
Stage	I: 15 (11%)	I: 271 (22%)	p = 0.003
	II: 48 (33%)	II: 421 (35%)	
	III: 48 (33%)	III: 328 (27%)	
	IV: 33 (23%)	IV: 194 (16%)	
Grade	1 or 2: 40 (28%)	1 or 2: 849 (70%)	p < 0.001
	3: 104 (72%)	3: 365 (30%)	
	**MAC**	**NMAC**	
Stage by site	RIGHT	RIGHT	p = 0.38
	I: 10 (11%)	I: 96 (17%)	
	II: 34 (37%)	II: 218 (38%)	
	III: 29 (31%)	III: 164 (29%)	
	IV: 19 (21%)	IV: 91 (16%)	
	LEFT	LEFT	
	I: 5 (10%)	I: 175 (27%)	p = 0.008
	II: 14 (27%)	II: 203 (32%)	
	III: 19 (37%)	III: 164 (25%)	
	IV: 14 (27%)	IV: 103 (16%)	
	Right vs Left	Right vs Left	
	p = 0.60	p < 0.001	

MAC, Mucinous adenocarcinomas; NMAC, Non-mucinous adenocarcinomas.

### Multivariate model, all patients

All variables with a correlation coefficient >0.5 with stage or grade were dropped from the final model that included gender, age, stage, grade, laterality, and mucinous histology. The results of the multivariate analysis are reported in Table 3; the proportional Hazard assumption was not violated (data not shown). Male sex, right side and stage maintained an independent effect on OS. Mucinous histology was not an independent prognostic factor, indicating that its effect likely depends on proximal location and higher grade and stage at diagnosis.

**Table 3:** Multivariate analysis of overall survival.

	P	HR	95.0% CI	
			Lower	Upper
Gender (ref: Male)	0.12	0.87	0.73	1.04
Age	<0.001	1.064	1.054	1.073
Side (Ref: Left)	0.02	1.23	1.03	1.47
Histology (Ref: NMAC)	0.81	1.03	0.79	1.34
Stage (p-trend)	<0.001			
Stage (I vs II)	0.002	1.71	1.21	2.41
Stage (I vs III)	<0.001	3.22	2.30	4.50
Stage (I vs IV)	<0.001	11.56	8.26	16.20
Grade (Ref:1/2)	0.007	1.28	1.07	1.53

MAC, Mucinous adenocarcinomas; NMAC, Non-mucinous adenocarcinomas.

The effect of stage on tumor side was further tested by propensity score matching. The analysis showed that the effect of side on prognosis was mainly restricted to advanced stage tumors. More specifically, the prognosis of stage I and II right- and left-sided CRCs was similar (HR = 0.69 [0.35–1.35], p = 0.28 and HR = 0.85 [0.59–1.23], p = 0.40, respectively), stage III right-sided tumors showed a trend towards poorer OS (HR = 1.34 [0.98–1.85], p = 0.07), whereas stage IV right-sided CRCs had a significantly worse outcome (HR = 1.55 [1.13–2.12], p = 0.005). Overall, 49 (44%) stage IV right-sided colon cancer patients received first-line chemotherapy against 69 (59%) stage IV left-sided colon cancer patients.

### Multivariate models, patients who relapsed and who received first-line chemotherapy

**Figure 2:**
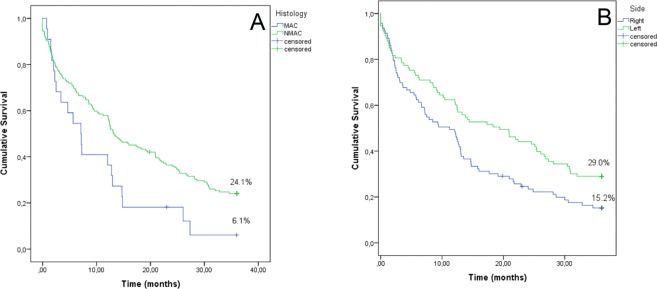
(**A**) Overall survival by histology in relapsed patients. Blue = mucinous carcinoma, green = non-mucinous tumor. (**B**) Overall survival by tumor side in relapsed patients. Blue = right side, green = left side.

Overall, 187 patients relapsed at a mean time after diagnosis of 16.4 (9.2–27.4) months. There were no differences in the relapse rate and the number of metastatic sites according to laterality and histology (Table 4). By univariate survival analysis, both mucinous histology (median OS 7.1 vs 13.1 months, p = 0.054; Fig. 2A) and right side (median OS 11.2 vs 19.4 months, p = 0.003; Fig. 2B) were significantly associated with OS. The effect on OS was then calculated on a multivariable model that included sex, age, laterality, mucinous histology, number of metastatic sites, administration of chemotherapy, adding a time-dependent covariate accounting for the non-parallel hazard of this last variable (Table 5). Age, laterality, number of metastatic sites and chemotherapy were found to be independent prognostic factors for OS. Mucinous histology was close to the significance (p = 0.059), however, the number of MACs was too limited (Table 1) to draw definitive conclusions.

**Table 4:** Relapse rate according to side, histology and number of metastatic sites.

	Side		Histology	
	Right	Left	MAC	NMAC
Relapsed (%)	93/661 (14%)	94/697 (13%)	22/144 (15%)	165/1214 (14%)
**Number of metastatic sites (%)**				
1	50/661 (8%)	55/697 (8%)	12/144 (8%)	93/1214 (8%)
>1	43/661 (6%)	39/697 (6%)	10/144 (7%)	72/1214 (6%)

MAC, Mucinous adenocarcinomas; NMAC, Non-mucinous adenocarcinomas.

**Table 5:** Multivariate analysis of overall survival in relapsed patients.

	Univariate Cox’s regression Model		Multivariate Model	
	P value	HR (95% CI)	P value	HR (95% CI)
Gender (Ref: Male)	0.677	1.08 (0.76–1.52)	0.093	1.35 (0.95–1.90)
Age	<0.001	1.03 (1.01–1.05)	0.003	1.03 (1.01–1.05)
Side (Ref: Left)	0.023	1.48 (1.05–2.07)	0.043	1.41 (1.01–1.97)
Histology (Ref: NMAC)	0.059	1.63 (0.98–2.72)	0.055	1.61 (0.99–2.62)
Number of metastatic sites (Ref: 1)	0.026	1.48 (1.05–2.09)	0.002	1.73 (1.22–2.45)
Chemotherapy (Ref: No)	0.017	0.05 (0.00–0.58)	<0.001	0.16 (0.06–0.42)
Log(Time)*Chemotherapy	0.036	5.97 (1.12–31.76)	0.003	3.77 (1.58–8.98)

MAC, Mucinous adenocarcinomas; NMAC, Non-mucinous adenocarcinomas.

**Figure 3:**
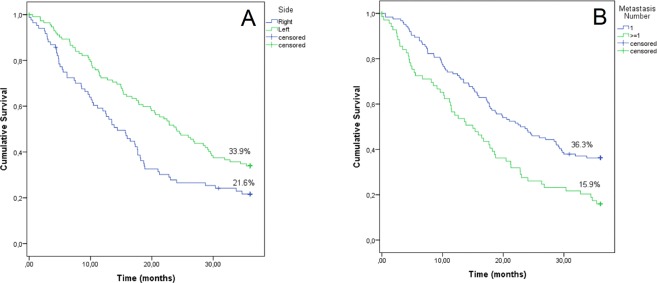
(**A**) Overall survival by tumor side in patients treated with first-line chemotherapy. Blue = right side, green = left side (p = 0.005). (**B**) Overall survival by number of metastatic sites in patients treated with first-line chemotherapy. Blue = 1 metastatic site, green ≥1 metastatic site (p = 0.001).

Finally, a subgroup of 197 out of 203 metastatic patients treated with first-line chemotherapy was analyzed (n = 6 were excluded as they had no valid data). Eighty-four patients (42.6%) had right-sided and 113 (57.4%) left-sided tumors; the median follow-up from relapse was 18.2 months. By univariate survival analysis (Table 6), female gender (p = 0.035, data not shown), right side (median survival 14.4 vs 24.0 months, p = 0.005; Fig. 3A), more than 1 metastatic site (median 15.0 vs 22.8 months, p = 0.001; Fig. 3B) and grade 3 (p = 0.028, data not shown) correlated with worse OS. Conversely, mucinous histology was not a significant prognostic factor. Age was only near significance (p = 0.075), but was maintained in the multivariate model. All five variables had a statistically significant independent effect by multivariate analysis (Table 6).

**Table 6:** Multivariate analysis of overall survival in patients receiving first line chemotherapy.

	Univariate Cox’s regression Model		Multivariate Model	
	P value	HR (95% CI)	P value	HR (95% CI)
Gender (Ref: Male)	0.035	1.45 (1.02–2.04)	0.006	1.68 (1.16–2.43)
Age	0.075	1.02 (1.00–1.04)	0.004	1.03 (1.01–1.05)
Side (Ref: Left)	0.005	1.61 (1.15–2.25)	0.014	1.53 (1.09–2.16)
Grade (Ref: 1 or 2)	0.029	1.45 (1.04–2.04)	0.032	1.47 (1.03–2.09)
Number of metastatic sites (Ref: 1)	0.001	1.79 (1.27–2.50)	0.002	1.73 (1.22–2.44)

## Discussion

The main results of the present study are that right side location is an independent prognostic factor in advanced CRC; this effect is mainly attributable to tumors with non-mucinous histology. Conversely, mucinous CRCs have a poor prognosis when relapsing regardless of the primary tumor-side. These data are consistent with the growing evidence from previous metanalysis^3^ and retrospective studies in more selected clinical settings^6,10–12^, and provide new evidence on the role of laterality and mucinous histology in patients with progressive disease.

The prognostic significance of right-sided CRC was recently reported by Yang *et al*.^4^ on a very large US cohort of nearly 58.000 patients from the Surveillance, Epidemiology, and End Results (SEER) database diagnosed from 2000 to 2012. More specifically, proximal location affected negatively the outcome in advanced stage of disease and in patients ≤70 years, and it was predictive of chemoresistance. Laterality was not related to age in our cohort, however, it was presented as a continuous variable and not categorically based on a 70 years cut-off^4^. The limited number of subjects limits any further assumption.

Our survival data were modeled by a multivariable analysis that accounted for acknowledged unfavorable prognostic factors and confirmed a higher risk of death for right-sided CRC patients limited to the subgroup with NMACs. Whether improvements in the genetic classification of CRCs will surpass the gross distinction between right and left-sided cancers is unknown^13,14^. So far, these categories that can be easily applied in any setting have resisted the challenge of molecular stratification.

Laterality has also shown a significant effect on response to treatment^4^. Left colon tumors treated with cetuximab in KRAS wild-type disease or bevacizumab experience prolonged PFS and a higher response rate than proximal colon cancers^12,27–29^. Furthermore, at least in the metastatic setting, the effect of tumor site on survival seems to be independent of RAS^10^ and BRAF status^6,11^. Indeed, molecular subtypes of CRCs are differentially distributed between proximal and distal sites (reviewed elsewhere)^30,31^, but there are not genetic changes specific of either location rather a gradient of frequency. Consistent with previous reports^9–12,27,28^, our data confirmed a worse clinical outcome for right-sided CRC patients treated with chemotherapy. Although, the number of metastatic sites was independently associated with survival and tumor stage distributions at diagnosis were different between right and left sides, we did not observe any difference in relapse rates or number of metastatic sites between right and left-sided tumors, further reinforcing the hypothesis that survival differences are more related to biological differences than to tumor burden. This has also been reported by Venook *et al*.^29^, who showed that laterality is independently prognostic when adjusted for metastatic sites. Unfortunately, in the present study from cancer registry-based population recorded between 2004 and 2009, mutational analysis of RAS and BRAF genes were performed only in a minority of cases insufficient for statistical analysis.

Several studies including a recent metanalysis have identified mucinous histology as a poor prognostic factor irrespective of stage at diagnosis^7,8,16–18^ and a predictor of poor response to chemotherapy^32,33^. In the study by Yang *et al*.^4^, mucinous histology correlated negatively with survival in left-sided CRCs and positively in right-sided tumors. This may reflect differences of molecular subtypes between proximal and distal MACs^20^. Thus, hypermutated tumors with MSI prevail in the right colon and have a better outcome than microsatellite-stable, BRAF-mutated left-sided MACs^30,34–36^. In our analysis, MACs prognosis was not affected by tumor side. However, upon recurrence, MACs showed a poorer prognosis than NMACs despite chemotherapy was given in the same percentage in both groups. Likewise, BRAF mutation and MSI do not predict tumor recurrence in early stage CRC, while they both are negative prognostic factors in advanced disease^36–38^.

Indeed, mucinous histology is a crude surrogate for underlying genetic changes as it is based on an arbitrary threshold of 50% extracellular mucin that is poorly reproducible as shown by the reclassification of 30% of original diagnoses in the present series. Also, signet-ring component, a variant of MAC with dismal prognosis, is not always reported and this might affect the comparison between different series. Furthermore, similar frequency of MSI, RAS and BRAF mutations have been reported in MACs and adenocarcinomas with a mucinous component (<50%) challenging current definition^15^.

The strong points of the present study are its population-based design, the limited period of accrual (2004–2009), the adequate length of observation (median, 66 months, IQ range, 25–79 months) and its mono-institutional setting. However, we were not able to model disease-specific survival and disease-free survival. Furthermore, compared with studies performed at tertiary referral centers or clinical trials, the present study is limited by less accurate follow-up data, lack of molecular analysis, and more variable treatments. Nevertheless, the median survival of our patients treated with first-line chemotherapy was in line with previous data^2^, further reinforcing our results.

In conclusion, colon cancer laterality and mucinous histology are working categories that maintain a clinical relevance especially in the context of palliative treatments of advanced disease. They may be useful to stratify patients in clinical trials and can be exploited as useful indicators for molecular typing. Their field of action, however, is likely to progressively reduce as they give way to more detailed genetic pathological classifications.

## References

[1] Ferlay J (2015). Cancer incidence and mortality worldwide: sources, methods and major patterns in GLOBOCAN 2012. Int. J. Cancer..

[2] Brenner H, Kloor M, Pox CP (2014). Colorectal cancer. Lancet..

[3] Yahagi M, Okabayashi K, Hasegawa H, Tsuruta M, Kitagawa Y (2016). The Worse Prognosis of Right-Sided Compared with Left-Sided Colon Cancers: a Systematic Review and Meta-analysis. J. Gastrointest. Surg..

[4] Yang J (2016). Characteristics of Differently Located Colorectal Cancers Support Proximal and Distal Classification: A Population-Based Study of 57,847 Patients. PLoS. One..

[5] Lee GH (2015). Is right-sided colon cancer different to left-sided colorectal cancer? – a systematic review. Eur. J. Surg. Oncol..

[6] Missiaglia E (2014). Distal and proximal colon cancers differ in terms of molecular, pathological, and clinical features. Ann. Oncol..

[7] Langner C (2012). Mucinous differentiation in colorectal cancer—indicator of poor prognosis?. Histopathology..

[8] Verhulst J, Ferdinande L, Demetter P, Ceelen W (2012). Mucinous subtype as prognostic factor in colorectal cancer: a systematic review and meta-analysis. J. Clin. Pathol..

[9] Price TJ (2015). Does the primary site of colorectal cancer impact outcomes for patients with metastatic disease?. Cancer..

[10] Brulé SY (2015). Location of colon cancer (right-sided versus left-sided) as a prognostic factor and a predictor of benefit from cetuximab in NCIC CO.17. Eur. J. Cancer..

[11] Loupakis, F. *et al*. Primary tumor location as a prognostic factor in metastatic colorectal cancer. *J*. *Natl*. *Cancer*. *Inst*. **107**, 10.1093/jnci/dju427 (2015). 10.1093/jnci/dju427 PMC4565528 25713148

[12] Venook, A. P. *et al*. Impact of primary (1°) tumor location on overall survival (OS) and progression-free survival (PFS) in patients (pts) with metastatic colorectal cancer (mCRC): Analysis of CALGB/SWOG 80405 (Alliance). *J*. *Clin*. *Oncol*. 34 (suppl; abstr 3504) (2016).

[13] The Cancer Genome Atlas Network (326 collaborators) (2012). Comprehensive molecular characterization of human colon and rectal cancer. Nature..

[14] Guinney J (2015). The consensus molecular subtypes of colorectal cancer. Nat. Med..

[15] Bosman, F. T., Carneiro, F., Hruban, R. H. & Theise, N. D. WHO classification of tumours of the digestive system *4th ed*. *International Agency for Research on Cancer**(*2010).

[16] Hugen N, van Beek JJ, de Wilt JH, Nagtegaal ID (2014). Insight into mucinous colorectal carcinoma: clues from etiology. Ann. Surg. Oncol..

[17] Farhat MH (2008). Effect of mucin production on survival in colorectal cancer: a case-control study. World. J. Gastroenterol..

[18] Xie L, Villeneuve PJ, Shaw A (2009). Survival of patients diagnosed with either colorectal mucinous or non-mucinous adenocarcinoma: a population-based study in Canada. Int. J. Oncol..

[19] Kim SH (2013). Prognostic value of mucinous histology depends on microsatellite instability status in patients with stage III colon cancer treated with adjuvant FOLFOX chemotherapy: a retrospective cohort study. Ann. Surg. Oncol..

[20] Leopoldo S (2008). Two subtypes of mucinous adenocarcinoma of the colorectum: clinicopathological and genetic features. Ann. Surg. Oncol..

[21] Liu XP (2004). Two subtypes of mucinous colorectal carcinoma characterized by laser scanning cytometry and comparative genomic hybridization. Int. J. Oncol..

[22] Tournigand C (2004). FOLFIRI followed by FOLFOX6 or the reverse sequence in advanced colorectal cancer: a randomized GERCOR study. J. Clin. Oncol..

[23] Cassidy J (2004). XELOX (capecitabine plus oxaliplatin): active first-line therapy for patients with metastaticcolorectal cancer. J. Clin. Oncol..

[24] Grothey A, Sargent D, Goldberg RM, Schmoll HJ (2004). Survival of patients with advanced colorectal cancer improves with the availability of fluorouracil-leucovorin, irinotecan, and oxaliplatin in the course of treatment. J. Clin. Oncol..

[25] www.NCCN.org/patients Version I (2017).

[26] Eisenhauer EA (2009). New response evaluation criteria in solid tumor: revised RECIST guideline (version 1.1). Eur. J. Cancer..

[27] Tejpar S (2017). Prognostic and predictive relevance of primary tumor location in patients with RAS wild-type metastatic colorectal cancer: Retrospective analyses of the CRYSTAL and FIRE-3 trials. JAMA. Oncol..

[28] Petrelli F (2017). Prognostic survival associated with left-sided vs right-sided colon cancer. JAMA. Oncol..

[29] Venook, A. P. *et al*. Primary (1°) tumor location as an independent prognostic marker from molecular features for overall survival (OS) in patients (pts) with metastatic colorectal cancer (mCRC): Analysis of CALGB/SWOG 80405 (Alliance). *J*. *Clin*. *Oncol*. **35** (suppl; abstr 3503) (2017).

[30] Müller MF, Ibrahim AE, Arends MJ (2016). Molecular pathological classification of colorectal cancer. Virchows. Arch..

[31] Yamauchi M (2012). Assessment of colorectal cancer molecular features along bowel subsites challenges the conception of distinct dichotomy of proximal versus distal colorectum. Gut..

[32] Negri FV (2005). Mucinous histology predicts for reduced fluorouracil responsiveness and survival in advanced colorectal cancer. Ann. Oncol..

[33] Catalano V (2009). Mucinous histology predicts for poor response rate and overall survival of patients with colorectal cancer and treated with first-line oxaliplatin- and/or irinotecan-based chemotherapy. Br. J. Cancer..

[34] Wang MJ (2015). Prognostic Significance and Molecular Features of Colorectal Mucinous Adenocarcinomas: A Strobe-Compliant Study. Medicine. (Baltimore).

[35] Samowitz WS (2005). Poor survival associated with the BRAF V600E mutation in microsatellite-stable colon cancers. Cancer. Res..

[36] Tran B (2011). Impact of BRAF mutation and microsatellite instability on the pattern of metastatic spread and prognosis in metastatic colorectal cancer. Cancer..

[37] Roth AD (2010). Prognostic role of KRAS and BRAF in stage II and III resected colon cancer:results of the translational study on the PETACC-3,EORTC 40993, SAKK 60-00 trial. J. Clin. Oncol..

[38] Innocenti, F. *et al*. Somatic DNA mutations, MSI status, mutational load (ML): Association with overall survival (OS) in patients (pts) with metastatic colorectal cancer (mCRC) of CALGB/SWOG 80405 (Alliance). *J*. *Clin*. *Oncol*. **35** (suppl; abstr 3504) (2017).

